# Novel self-assembling peptide for management of bleeding associated with colorectal angiodysplasia

**DOI:** 10.1055/a-2313-3786

**Published:** 2024-05-17

**Authors:** Kenichiro Okimoto, Tomoaki Matsumura, Naoki Akizue, Yuki Ohta, Takashi Taida, Jun Kato, Naoya Kato

**Affiliations:** 1 Gastroenterology, Graduate School of Medicine, Chiba University, Chiba, Japan; 2 Gastroenterology, Graduate School of Medicine, Chiba University, Chiba, Japan; 3 92154 Endoscopy Center, Chiba University Hospital, Chiba, Japan

Angiodysplasia is characterized by the presence of abnormal, ectatic, dilated, tortuous, and typically small (<10 mm) blood vessels within the gut 1 . Argon plasma coagulation is a commonly performed treatment for angiodysplasia that has proven to be effective 2 ; however, perforations have been reported with this treatment 3 .

PuraStat (3D-Matrix Europe, Caluire, France) represents an innovative synthetic self-assembling peptide system approved for achieving hemostasis. Upon contact with blood, its unique transparent gel formulation undergoes activation due to a pH change, forming an extracellular scaffold matrix. This matrix acts as a durable mechanical barrier at the bleeding site, facilitating intrinsic hemostasis in vivo. In the context of endoscopic submucosal dissection, PuraStat has been reported to be effective for managing bleeding in conditions such as gastric antral vascular ectasia 4 5 . Here we report a case in which the application of PuraStat was effective in controlling bleeding from angiodysplasia ( Video 1 ).

PuraStat was applied to control bleeding from angiodysplasia in the cecum.Video 1

The patient was an 85-year-old woman with a history of aortic regurgitation who underwent aortic valve replacement. She was on 15 mg edoxaban tosilate hydrate and 2.5 mg prednisolone. Fecal occult blood test was conducted due to chronic anemia, which revealed a positive result. Subsequent lower gastrointestinal endoscopy revealed angiodysplasia with blood oozing into the cecum ( Fig. 1 ). Hemostasis was achieved by applying PuraStat to the bleeding site ( Fig. 2 ). Although most of the PuraStat was accidentally peeled off upon contact with blood, a small amount remained in contact with the bleeding site, which successfully maintained hemostasis ( Fig. 3 ). Additional PuraStat was applied for reinforcement before concluding the procedure ( Fig. 4 ). Thereafter, there was no recurrence of bleeding, and the hemoglobin level increased from 7.7 g/dL prior to hemostasis to 9.5 g/dL with only the administration of oral iron. Thus, we found that PuraStat is convenient to apply and effective for achieving hemostasis in colorectal angiodysplasia, without any associated tissue damage.

**Fig. 1:**
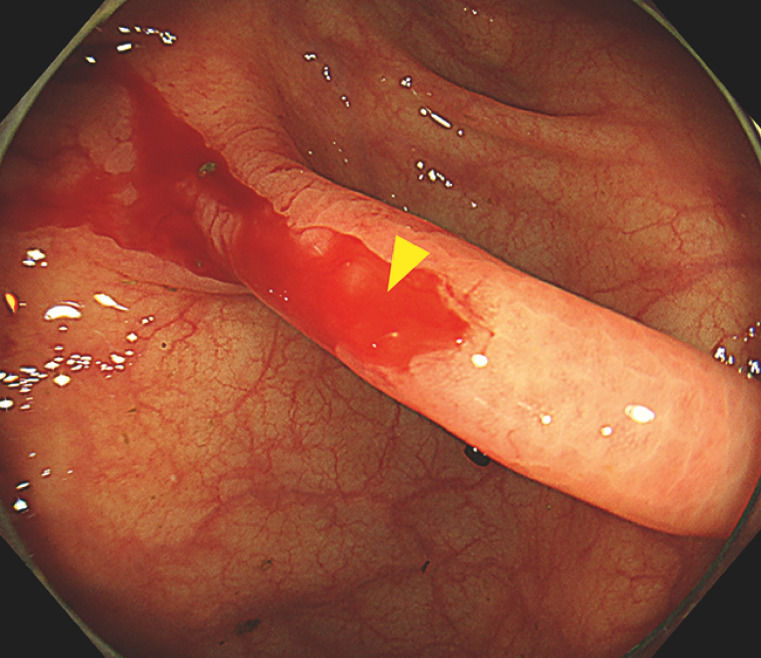
Lower gastrointestinal endoscopy revealed angiodysplasia with blood oozing (arrowhead) into the cecum.

**Fig. 2:**
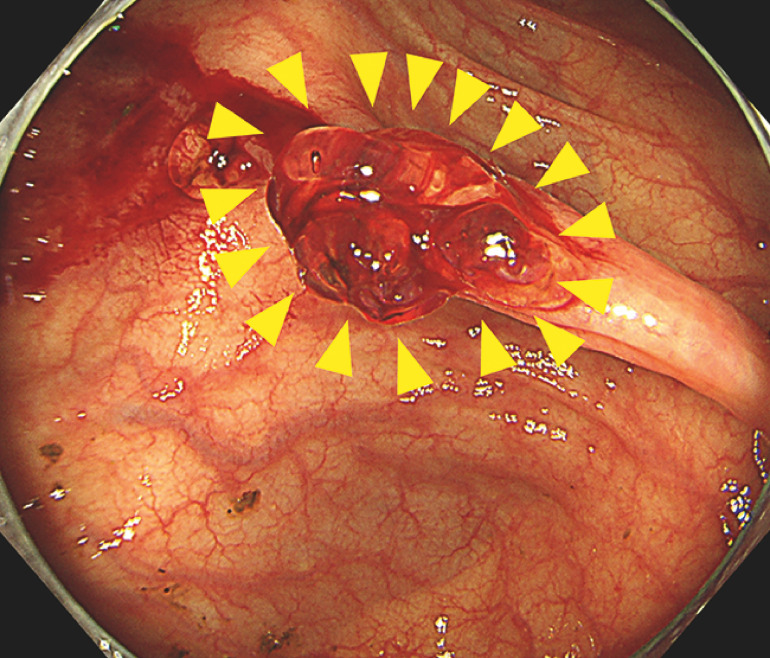
Hemostasis was achieved by applying PuraStat to the bleeding site (arrowheads).

**Fig. 3:**
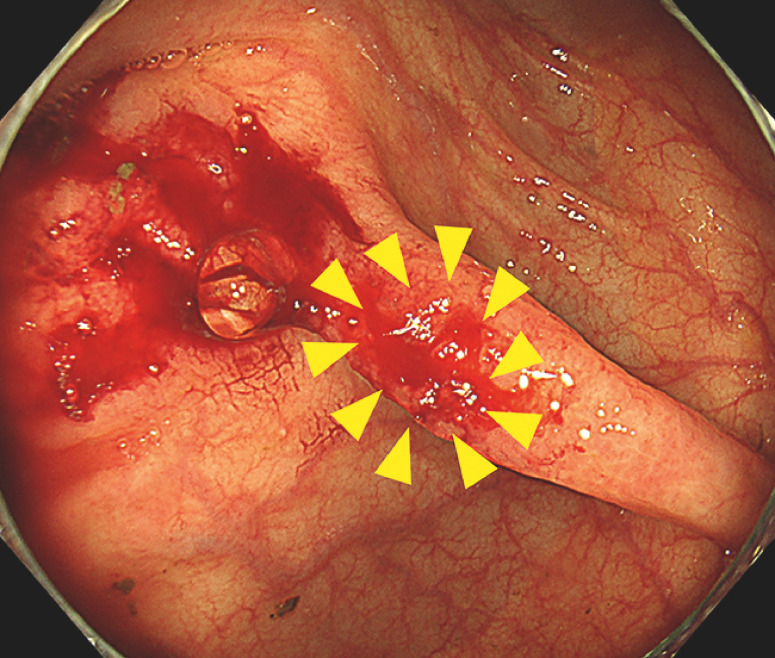
Although PuraStat was accidentally peeled off upon contact with blood, a small amount of PuraStat remained in contact with the bleeding site (arrowheads), which maintained hemostasis.

**Fig. 4:**
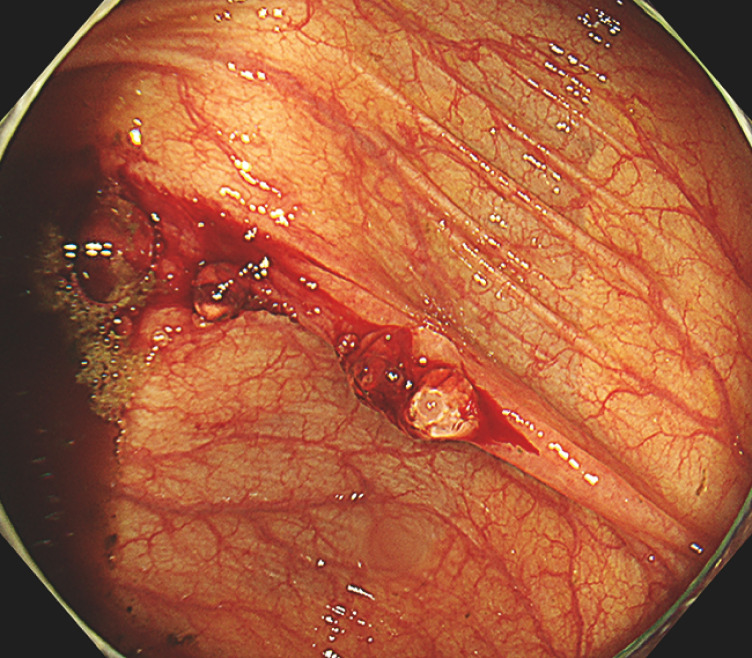
Additional PuraStat was applied for reinforcement.

Endoscopy_UCTN_Code_TTT_1AQ_2AZ

## Notes

Endoscopy E-Videos https://eref.thieme.de/e-videos
*E-Videos* is an open access online section of the journal *Endoscopy* , reporting on interesting cases and new techniques in gastroenterological endoscopy. All papers include a high-quality video and are published with a Creative Commons CC-BY license. Endoscopy E-Videos qualify for HINARI discounts and waivers and eligibility is automatically checked during the submission process. We grant 100% waivers to articles whose corresponding authors are based in Group A countries and 50% waivers to those who are based in Group B countries as classified by Research4Life (see: https://www.research4life.org/access/eligibility/ ). This section has its own submission website at https://mc.manuscriptcentral.com/e-videos .
